# Achievements and challenges in health management for aged individuals in primary health care sectors: a survey in Southwest China

**DOI:** 10.1186/s12889-020-8210-2

**Published:** 2020-03-17

**Authors:** Li Li, Rui Zhang, Yong Chen, Haoyue Deng, Shili Liu, Geng Wang, Mei Wang, Shengxiang Liang, Wei Xing, Hai Lin, Ying Li

**Affiliations:** 1 Department of Social Medicine and Health Service Management, Army Medical University (Third Military Medical University), No.30 Gaotanyan Road, Shapingba District, Chongqing, 400038 China; 2 Chongqing City Management College, Chongqing, China

**Keywords:** Health management, Aged individuals, Basic public health service, Lay healthcare worker, China

## Abstract

**Background:**

China has rapidly transformed into an ageing nation and will be one of the countries with the highest percentage of aged people in 2050. Healthcare management for the aged (HMA) in basic public health service (BPHS), which is delivered by lay healthcare workers (LHWs) in primary health care (PHC) sectors, is an important strategy to address the healthcare challenges that have resulted from ageing in China since 2009. This survey aimed to understand the achievements made and challenges faced by HMA in Southwest China.

**Methods:**

A multilevel stratified random and consecutive sampling method was used to select study places and participants respectively, and mixed research methods were used to collect data from the aged individuals, LHWs and leaders in PHC sectors. SPSS 21.0 was used for data analysis.

**Results:**

Seven hundred seventy-two surveys with aged people (over 60 years old), 16 focus group discussions (FGDs) with 96 aged people, and 32 in-depth interviews with 16 LHWs and 16 leaders were completed in PHC sectors. More than 85% of aged individuals had knowledge and utilization of HMA, and over 94% of these respondents were satisfied with HMA. Meanwhile, challenges in HMA delivery included weakness (unmet items and lack of appropriate assessment indicators) in HMA design, low capacity of PHC sectors and competency of LHWs to deliver HMA, poor health literacy of aged individuals, insufficient funds and a lack of multi-sector cooperation.

**Conclusions:**

Though significant achievements in HMA were observed, this study highlighted the challenges in further quality improvement of HMA delivery program in Southwest China. The “older-person-centered and integrated care” model provided a good theory to improve the quality of HMA by reinforcing the needs-based HMA design, building a comprehensive assessment strategy, improving the capacity of PHC sectors and the LHWs’ competency, and strengthening multi-sector cooperation.

## Background

In 2015, World Health Organization (WHO) reports on ageing and health reported that the population of ageing was rapidly increasing worldwide [1]. With 10% of its population being over 60 years of age, China quickly transformed into an ageing nation in 1999. The doubling (from 7 to 14%) of the proportion of people aged 65 years and older was accomplished just in 26 years in China [2]. By the end of 2017, the ageing population in China had increased to 17.4% [3]. The speed and scale of the ageing population are unprecedented all over the world. By the year 2050, it is predicted that 337 to 400 million of the Chinese population will be aged 65+ (about 23.9 to 26.9% of the total population) and that 107 to 150 million will be aged 80+ [4, 5]. Accordingly, China will be one of the countries in the world with the highest percentage of aged people in 2050. Ageing has therefore become a pervasive social problem in China as the Chinese “become aged before rich”, the lack of preparation for an aging society, specifically planning for the health needs of the elderly in China, challenges the health and social care system of this country [4].

The rapidly growing population of aged individuals brings great challenges to the health care system, for example, a surge of age-associated diseases encompassing chronic non-communicable diseases (NCDs) and mental health disorders [6]. Disease prevention, early diagnosis and maintenance of a healthy lifestyle through primary health care (PHC) are very important to address those health challenges among aged individuals [6]. Thus, health management for aged individuals has become a promising strategy [7]. In response to the accelerating ageing population, WHO put forth goals of healthy ageing in the world report on ageing and health in 2015 [1]; China launched BPHS incorporating HMA explicitly in 2009 [3, 8, 9] and quickly undertook the development of aged care service into the 13th five-year plan in the middle of 2015 [3]. Basic public health services (BPHS) for all residents is one of the priorities of the new health reform in China which was launched in 2009. Since 2009, the national BPHS programs have been widely carried out across primary health care (PHC) sectors including community health centers (CHCs) and stations in urban areas, township hospital centers (THCs) and village clinics in rural areas of China. These play an important role in ensuring and improving the health condition of residents, and the equity and accessibility of public health have greatly improved [10, 11]. Though the content of BPHS had been modified three times by 2015, HMA was always listed as one of the core programs of BPHS [12]. All items of HMA in BPHS are delivered by LHWs in primary health care sectors (PHCs).PHC sector, mainly provided and funded by the government in China, was a sector provided primary health care service for the residents who lived in the area of PHC sector, including disease control, management of chronic disease, health promotion and education. Free HMA for aged individuals (people aged 65+) included four items: lifestyle evaluation and health assessment (LE and HA), health check-up (HC) (such as measuring height, weight, waist circumference, vision tests, heart rate, and blood pressure), auxiliary exam (AE) (such as fasting-blood glucose test, electrocardiograms, and B-scan ultrasounds) and health guidance (HG) (such as disease prevention, self-care and injury prevention, self-rescue) [12].

Few studies have reported one or two aspects (satisfaction/needs/knowledge/utilization, separately) on the current situation of HMA, most of which have focused on investigating rate of the aged population using HMA or satisfaction to HMA [13–26]; some studies have assessed the situation of HMA delivery by LHWs in PHC [27, 28], while others have revealed that both quantity and quality of LHWs in PHC sectors need supplementation and improvement [29–36], with some basic hardware facilities required to have replenishment as well [32–34]. Additionally, a study by Hu XY [30] indicated the lack of a unified and quantifiable performance evaluation index system. However, there is no systematic assessment of HMA delivery in primary health care (PHC) sectors [10]. Furthermore, a national assessment of BPHS delivery in PHC sectors has only been performed once in 2010, indicating that Southwest China lags behind Central and Eastern China in this aspect [37].

Therefore, this study selected Southwest China as the study region and aimed to provide a preliminary evaluation of the achievements of HMA and the associated factors, and assessment of the challenges faced by HMA.

## Methods

This cross-sectional survey utilized mixed research methods to collect data from February 2015 to August 2016. Both quantitative and qualitative research methods were used to evaluate the delivery of HMA in PHC sectors from both aged individuals and providers (LHWs and leaders from PHC sectors).

### Study setting

In Southwest China, we purposively selected Chongqing municipality (as a region with more developed socio-economic development, with the Gross Domestic Product (GDP) at 2.04 trillion RMB and per capita GDP at 66.2 thousand RMB in Chongqing.) and Guizhou province (as a region with less developed socio-economic conditions, with 1.48 trillion RMB in Guizhou, per capita GDP was 41.4 thousand RMB in Guizhou) as the study regions [38]. Southwest China is an underdeveloped area in China, whose per capita net income is much lower than that of Eastern and Central China [33].

A multi-stage random sampling was used and we selected one county/district to represent the socio-economic development of Guizhou and Chongqing respectively. Then the PHC sectors in the selected county/district were divided into THCs (in rural areas) and CHCs (in urban areas). According to the quality of BPHS delivery in previous year, THCs and CHCs were divided into developed and less developed groups for the sample representation and comprehensive understanding of the status of HMA. Finally, four THCs and four CHCs were random selected from each province, two THCs/CHCs that were more developed and another two less developed. In total, eight THCs and eight CHCs were chosen as the final study communities. The flow chart of study region selection was shown in Appendix A.

Chongqing transformed into an ageing society in 1994, which was 5 years earlier than China as a whole. The sixth census indicated that people aged 65+ comprised 11.56% of the population in Chongqing, which was much higher than the national level (8.87%), and this value became the highest among all provinces and regions in China [37]. It is predicted that the estimated percentage of individuals aged 65+ will reach 28.15% by 2050 [39]. Guizhou rapidly transformed into an ageing society in 2003; the percentage of individuals 65+ reached 8.71% in 2010 and will reach up to 21% by 2050 [40]. However, the underdeveloped socio-economy cannot provide health security for aged individuals, and self-support is very weak in Guizhou province.

BPHS has been carried out since 2009 in Chongqing and 2010 in Guizhou. Recently, PHC sectors in Chongqing have developed quickly; almost all PHC sectors are held by the government [41] and have enough buildings (even more than required by the central government) [42]. However, all PHC sectors are not only short of LHWs in number, but also lacked LHWs qualified for BPHS in terms of education background, skills and professional titles (approximately 70% of LHWs have only an education of college level or below either with a primary professional title or with no title) [40]. PHC sectors are even less developed in Guizhou compared with those in Chongqing [43, 44]. In China, less than 20% of CHCs in urban areas are held and funded by the government, almost 60% of CHCs have to use the rented buildings, and only 31.5% of CHCs meet the infrastructure requirements to provide PHC; also, 58% of LHWs attain a secondary school education or below and 70% of LHWs have only a primary professional title or no title [43].

### Study participants and data collection

#### Quantitative research

We used consecutive sampling method to recruit participants. All people who showed up in the selected THCs/CHCs, met inclusion criteria and were interested in our study during our study period were recruited as participants. The inclusion criteria included the following: (1) people aged over 60 years old; and (2) those who are willing to participate in the survey. The exclusion criteria included the following: (1) those who were diagnosed with a mental illness or had disturbed consciousness; (2) those who had difficulties with speech or hearing; and (3) those who declined to participate in the survey. All participants completed an informed consent form. A total of 772 aged people was included in questionnaire surveys.

A structured questionnaire with 4 sections was adopted to collect the data, including socio-demographic information (age, gender, height, weight, education, residence, occupation before retirement, health insurance, and distance to PHC sectors), knowledge about HMA, utilization of HMA and satisfaction with HMA. Questionnaire was designed by our research team through reviewing the existing literature reports and consulting experts before pilot study. Then, the questionnaire was pilot tested with 100 participants. All questionnaires were executed by trained investigators from our research group and the completed questionnaires were checked and examined by trained investigators for quality control.

#### Qualitative research

Focus group discussions (FGD) were conducted to determine the actual needs and identify the barriers to HMA delivery from the aged individuals; the aged people for the FGDs were purposively selected with the help of PHC sectors in the study region. The aim and procedures of the study were explained to and agreed by the participants though signing a consent form. We convened 16 FGDs comprised of 8 male and 8 female groups. In-depth interviews were utilized to explore barriers on the delivery of HMA from LHWs who provide HMA to aged individuals and leaders who are responsible for BPHS; 32 in-depth interviews with 16 LHWs and 16 leaders from the department of public health in PHC sectors enrolled in our study were purposively selected.

A semi-structured topic guide was used for all interviews. The Practical Robust Implementation and Sustainability Model (PRISM) [45], widely used as a theoretical framework in implementation research [46, 47], was used to guide the topic design. With the guide of the PRISM, this study collected data on barriers to HMA delivery in the following aspects: HMA design (interventions), PHC sectors’ characteristics and aged residents (recipients), cooperation across related institutions (external environment), and PHC infrastructure for HMA (organizational implementation and sustainability of the infrastructure). All interviews and FGDs were conducted in Mandarin in local meeting rooms. Three senior researchers (LiLi, Shili Liu, and Mei Wang) conducted all the interviews and FGDs. Each interview lasted approximately 40–60 min, and each FGD lasted 1–1.5 h. All interviews and FGDs were audio-recorded with consent of the participants and professionally transcribed for analysis.

### Data analysis

#### Quantitative analysis

Epi Data 3.1 was used to enter data, and Statistical Package for Social Science (SPSS 21.0 (IBM Corporation, Armonk, NY, USA)) was used for data analysis. A two-tailed probability level of *p* < 0.05 was chosen as the level of statistical significance. Missing data were excluded from the analysis. Descriptive statistics were used to describe study participants’ characteristics, knowledge about HMA, utilization of HMA and satisfaction with HMA provision. Factors associated with knowledge, utilization and satisfaction screened by the Chi-square test (*p* < 0.05) (Appendix B) were entered into multivariate logistic regression models (having no knowledge of HMA = 0, no use of HMA = 0, dissatisfaction with HMA = 0; having knowledge of HMA = 1, use of HMA = 1, satisfaction with HMA = 1), which were used to examine the effects of those factors on knowledge, utilization and satisfaction.

#### Qualitative analysis

The framework approach [48, 49] was used to analyze all qualitative data following a five-step process: familiarizing, indexing each transcript with a framework, summarizing data in an analytical framework, data synthesis and interpretation of data [48, 50]. Following the framework approach, all interviews were transcribed into a Word document, and then all transcripts were coded and classified. We generated themes on barriers for each part of the PRISM. The names of all the participants in the interviews were removed from the quotations of the results to preserve anonymity.

## Results

### Demographic characteristics of participants

A total of 772 aged people was included in the questionnaire survey (Table 1). Among them, more than half (56.9%) were aged 60–69 years old, 62.1% were female, 71.1% were aged people from Chongqing, 61.4% were urban residents and 75% were married. Meanwhile, 63.1% received HMA from THCs, and 56.3% received HMA from PHC with better quality. Among the respondents, 62.9% had only primary school education or no education, and more than 1/3 were peasant farmers. Basic health insurance covered almost all of the aged individuals, while nearly 80% lived close to PHC sectors (less than 1 km). Notably, close to 30% of the respondents were overweight, and 5.9% were obese. In addition, almost 30% of aged people self-reported unwell health, and 78.5% endured chronic non-infectious disease including hypertension, diabetes, heart disease, arthritis, cataracts, etc.

**Table 1:** Demographic characteristics of the participants for quantitative study

Demographic characteristics	Number	Percentage
Age(*n* = 772)		
60–69	439	56.9
70–80	273	35.4
≥ 80	60	7.8
Gender (*n* = 771)		
Male	292	37.9
Female	479	62.1
Residence(*n* = 765)		
Rural	295	38.6
Urban	470	61.4
Type of PHCs (*n* = 772)		
THCs	487	63.1
CHCs	285	36.9
Quality of PHCs (*n* = 772)		
Good	435	56.3
Poor	337	43.7
Region(*n* = 772)		
Chongqing	549	71.1
Guizhou	223	28.9
Marital status (*n* = 767)		
Married	575	75.0
Divorced/Widowed	192	25.0
Education (*n* = 771)		
Primary and below	485	62.9
Middle school	188	24.4
College and above	98	12.7
Occupation (*n* = 765)		
Employed in enterprises/institutions/government	276	36.1
Peasants/ rural migrant workers	283	37.0
Others	206	26.9
Health insurance(*n* = 771)		
Basic health insurance	757	98.2
Others	14	1.8
Distance to PHCs (*n* = 767)		
< 1 km	604	78.7
1-2 km	89	11.6
≥ 2 km	74	9.6
BMI (kg/m^2^, *n* = 758)		
<18.5	32	4.2
18.5–24.0	456	60.2
24.0–30.0	225	29.7
≥ 30.0	45	5.9
Self reported health (*n* = 771)		
Well	246	31.9
Not bad	296	38.4
Unwell	229	29.7
Chronic diseases (*n* = 772)		
Yes	606	78.5
No	166	21.5

PHCs refers to primary health care sector; THC refers to township health center; CHC refers to community health center; BMI refers to Body Mass index

Ninety-six aged people were divided into 16 FGD groups comprised of 8 male and 8 female groups. The participants were aged 60–90, with an average age of 71. Most of these participants had an education level of junior high school or below, and lived with their spouse. All of them had health insurance mainly consisting of a new rural cooperative medical system and health insurance for urban residents. Remarkably, most aged people reported chronic disease. Conditions such as age distribution, education degree, marital status, insurance category and chronic disease are shown in Table 2. Sixteen HCWs of 22–44 years old including 2 males and 14 females providing HMA program to the aged were interviewed; among them, 9 HCWs had a college education and 7 HCWs had middle school education; 5 HCWs have worked in this area for less than 5 years, and 8 HCWs for 5–15 years, 3 HCWs for 15 years and more. Sixteen leaders (9 males and 7 females) of 31–57 years old from the department of public health in PHC sectors enrolled in our study had in-depth interviews; Among them except for one leader only having education of middle school, the rest had a college education; 5 leaders have worked in this area for 5–15 years, 11leaders for 15 years and more.

**Table 2:** Characteristics of FGD Participants

Characteristics		Number
Gender	Male	48
	Female	48
Age	60–69	45
	70–79	38
	≥80	13
Education	Illiteracy	19
	Primary school	29
	Junior high school	25
	Senior high school and above	23
Marital status	Married	72
	Divorced	3
	Widowed	21
Health insurance	New rural cooperative health insurance	42
	Urban residents health insurance	40
	Urban worker health insurance	14
Chronic disease	None	12
	One type	38
	Two types and above	46

FGD refers to focus group discussion

### Knowledge, utilization of and satisfaction to HMA

We evaluated the knowledge, utilization of and satisfaction to HMA among the aged through questionnaire survey.

#### Knowledge

**Fig. 1:**
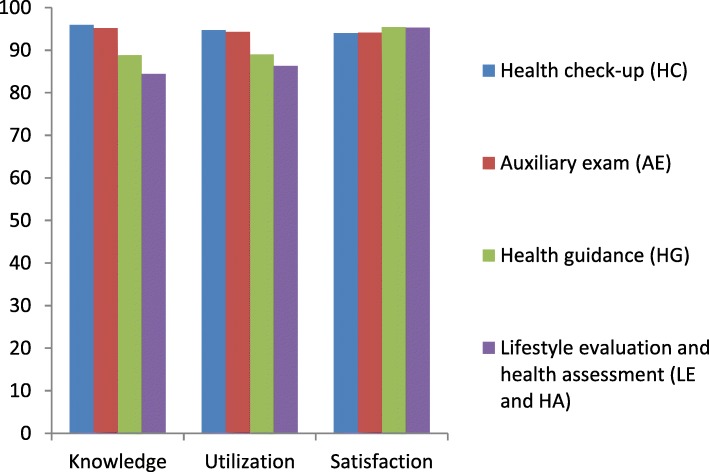
Knowledge and utilization of, and satisfaction with HMA. This figure presents the percentage of the aged had knowledge on and utilization of, and satisfaction with all healthcare management for the aged (HMA) items, including lifestyle evaluation and health assessment (LE and HA), health check-up (HC), auxiliary exam (AE) and health guidance (HG)

The knowledge of aged individuals for HC, AE, HG and LE and HA were 95.9, 95.2, 88.8, 84.4%, respectively (Fig. 1). Multivariate logistic regression (Table 3) showed that compared with Chongqing, aged people in Guizhou had less knowledge on LE and HA, as well as on HG. Aged people in PHCs with poor quality knew better about the four items of HMA. As for factor like occupation, peasant farmers had less knowledge about LE, HA and HG compared to people who employed in enterprises/institutions/government and others. Aged individuals with a BMI < 18.5 had less knowledge of LE and HA, while overweight aged people know more about LE and HA with a BMI increased.

**Table 3:** Logistic regression analysis on factors associated with knowledge and utilization of, and satisfaction to HMA

Variables	Knowledge				Utilization				Satisfaction			
	LE and HA	HC	AE	HG	LE and HA	HC	AE	HG	LE and HA	HC	AE	HG
Gender (*n* = 771)												
Male	–	–	–	–	–	–	–	–	1	1	–	1
Female	–	–	–	–	–	–	–	–	2.52 (1.11, 5.71)	2.10 (1.08, 4.06)	–	3.24 (1.42, 7.39)
Residence(*n* = 765)												
Rural	–	–	–	–	–	–	–	1	–	–	–	–
Urban	–	–	–	–	–	–	–	0.44 (0.21, 0.92)	–	–	–	–
Region(*n* = 772)												
Chongqing	1	–	–	1	1	–	–	1	–	–	–	–
Guizhou	0.33 (0.20, 0.54)	–	–	0.31 (0.18, 0.54)	0.19 (0.11,0.32)	–	–	0.13 (0.07, 0.24)	–	–	–	–
Type of PHCs (*n* = 772)												
THCs	–	–	–	–	–	–	–	–	–	–	1	–
CHCs	–	–	–	–	–	–	–	–	–	–	0.43 (0.22, 0.84)	–
Quality of PHCs (*n* = 772)												
Good	1	1	1	1	1	1	1	–	–	–	–	–
Poor	2.13 (1.29, 3.51)	2.33 (1.02, 5.31)	2.57 (1.19, 5.55)	2.04 (1.15, 3.61)	2.00 (1.12, 3.59)	2.33 (1.29, 3.51)	2.56 (1.25, 5.23)	–	–	–	–	–
Occupation (*n* = 765)												
Employed in enterprises/institutions/government	1	–	–	1	–	–	1	1	–	–	–	–
Peasants/ migrant workers	0.58 (0.34, 0.97)	–	–	0.52 (0.29, 0.92)	–	–	1.11 (0.56, 2.19)	0.41 (0.19, 0.89)	–	–	–	–
Others	1.44 (0.77, 2.71)	–	–	1.65 (0.78, 3.52)	–	–	4.03 (1.34, 12.13)	1.38 (0.60, 3.20)	–	–	–	–
BMI(*n* = 758)												
18.5–24.0	1	–	–	–	1	–	–	–	–	–	–	–
< 18.5	0.41 (0.18, 0.94)	–	–	–	0.33 (0.14, 0.81)	–	–	–	–	–	–	–
24.0–30.0	1.11 (0.68, 1.81)	–	–	–	1.09 (0.64, 1.88)	–	–	–	–	–	–	–
≥ 30.0	3.44 (0.80, 14.90)	–	–	–	5.13 (0.67, 39.33)	–	–	–	–	–	–	–

Notes: LE and HA refers to lifestyle evaluation and health assessment; HC refers to health check-up; AE refers to auxiliary exam; HG refers to health guidance; PHCs refers to primary health care sector; CHCs refers to community health centers; THCs refers to township hospital centers.“---”refers to this variable was not included in the logistic model for this independent variable

#### Utilization

The utilization of aged individuals for HC, AE, HG, LE and HA were 94.7, 94.3, 89.0, 86.3%, respectively (Fig. 1). Multivariate logistic regression (Table 3) showed that urban residents had lower utilization of HG. Compared with Chongqing, aged people in Guizhou had less utilization of LE and HA, as well as of HG. Aged people in PHCs with poor level were more likely to use LE and HA, HC and AE. As for factor like occupation, peasant farmers had less utilization of HG, whereas they had higher utilization of AE. Aged individuals with a BMI < 18.5 had less utilization of LE and HA, while overweight aged people were more likely to use LE and HA with a BMI increased.

#### Satisfaction

The satisfaction of aged individuals for HC, AE, HG, LE and HA were 94.0, 94.1, 95.4, 95.3%, respectively (Fig. 1). Multivariate logistic regression (Table 3) showed that female aged people were more satisfied with LE and HA, HC and HG than male respondents. Aged people were less satisfied with AE delivered by CHCs compared with that delivered by THCs.

### Challenges in HMA delivery

All interviews and FGDs indicated that the PHC sectors in Guizhou delivered all HMA items (LE and HA, HC, AE and HG) for aged 65+ in Guizhou according to the national BPHS guideline. In Chongqing, both LHWs and leaders reported that the PHC sectors provided all HMA items to all people aged 60+ in Chongqing with funding support from the local government. In addition, more HC items (including Ultrasonic B and chest radiography) were provided free to all people aged 60+ in Chongqing. However, results of the in-depth interviews with both LHWs and leaders together with results from FGDs showed numerous challenges in HMA delivery, which are summarized in the 4 PRISM domains in Table 4.

**Table 4:** Results of qualitative study about barriers to carry out HMA management

Core PRISM domains	Themes	Results	Quotation
Interventions –HMA program design	HMA system	Majority LHWs and all leaders reported HC items in national guideline cannot meet various needs of the elderly. In Chongqing, LHWs also reported that though HC has increased several items with funding support from local government, the HC items cannot meet needs of the elderly. The elderly in FGDs did not satisfy with HC items.	Given the limited HC items provided by HMA, the elderly can’t take HC items according to their actual needs. Each elderly has different kinds of health problems and thus their needs for HC items are different *(LHWs on HMA in THC)*. The elderly reported that they can’t take HC items on their needs, though added chest radiography, they need more items like gastroscope, colonoscope and rheoencephalogram *(LHWs on HMA in CHC)* It can only be said that I am basically satisfied with HC items. I think it is not good enough because the items of HC are too less *(Male elderly, Shapingba Community, FGD).*
	Performance assessment	All LHWs responded that performance assessment mainly through reviewing the program form was inappropriate as LHWs had to pay more attention to complete the form for assessment. On the other hand, all LHWs reported that the assessment just using the rate of the elderly included in HMA program instead of the quality of HMA delivery. As the current policy for the elderly participating in HMA is optional, instead of compulsory, it is hard to reach the rate required by BPHS guideline.	There are many challenges, one is that it is hard for us to achieve the required the rate of HMA, for example, there are 6000 elderly in this area, at least 65% of them should take HC items according to the requirement because the policy for the elderly to take HMA is optional, but we may achieve just around 40% and cannot met the requirement which directly impacts on funding for HMA and our income *(LHWs on HMA in CHC)*. In fact, we are serious on delivering HMA for each elderly, however, the forms required us to fill for the elderly are too complicated and the assessment on our work strictly based on the forms, which leads us take a lot of time to fill the form instead of provide actual HMA for the elderly We hope to leave more time to do some actual service for the elderly *(LHWs on HAM in THC)*.
	Inadequate investment	Majority of LHWs and leaders reported the funds for HMA program were insufficient to meet the various needs of the elderly. Almost all LHWs were unsatisfied with the low salary. Most leaders and HCWs reported PHC sectors took the measure of “clinical bring up public health” to improve salary for LHWs who deliver BPHS including HMA (to improve salary for HCWs deliver BPHS by using the revenue from the clinic department in PHC sectors) and it is in urgent need of increasing investment.	Funding for HMA could just meet the basic needs for the HMA delivery at present; and there is short of funds to pay for health lecturers for HMA *(LHWs on HMA in THC)*. In certain, the funding is not as much as we think, and the government subsidies are not enough (*LHWs on HMA in THC)*. In our community, the department of clinic medicine has a considerable revenue, which help our public health get through difficulties in funding, that is to say, we use the revenue from the clinic medicine to supplement our the public health, to improve salary for HCWs deliver BPHS *(HMA) (Leader in CHC).* Truthfully, regard to the income, we are really not satisfied *(LHWs on HMA in CHC)*. To be honest, the clinic department in our THC has helped BPHS a lot in funds. It would be very difficult to deliver HMA and the whole BPHS if it relies on government subsidies only *(LHWs on HMA in THC)*.
Recipients	Poor health literacy	Vast majorities of LHWs agreed that the poor literacy of the elderly was reasons for them to participate in HMA program in actively as the elderly only would like to receive HC once they recognize it is important. Many LHWs reported that the elderly fear heavy disease burden and so they rejected to take diseases screening due to lack of health literacy. Notably, some aged people had difficulty to access HMA just because of the low literacy of their families.	As for the poor health literacy, the greatest difficulty is the misunderstanding on HMA and reluctance by the residents, which is a big challenge to deliver HMA *(LHWs on HMA in THC)*. Our work is difficult because some residents are lack of health literacy and they would come to check their blood pressure only after we call them twice or more, while some others are unwilling to come no matter we give them how many calls *(LHWs on HMA in THC)*. At present, lack of knowledge on HMA among residents is one of the challenges. It is not all of the elderly know HMA. Secondly, some elderly unwilling to take part in HMA, especially those in low income, they worry about the economic burden on solve the health problems which are identified by health check during participating HMA. They think it is good to let it on even they have any health problem *(LHWs on HMA in THC)*. I want to receive health check of HMA from our THC, but my daughter in law does not believe HMA, and she told me THCs would cheat me even I have no any health problem. Thus, I hesitate to take HMA *(Female elderly, Zhongliangzhen township, FGD).*
	Human resource deficiency in PHCs	All LHWs and leaders agreed that PHC sectors has shortage of adequate qualified LHWs to undertake HMA program and LHWs for HMA often undertook more than one BPHS program. In terms of the quality, majorities of the leaders and LHWs reported PHC sectors extremely lacked of general practitioners and the quality of current LHWs for HMA is not good enough; more than half were major in nursing with the primary title; they had difficult in delivering specific and individualized HG and also inability to do a comprehensive and systematic health needs assessment for the elderly.	THCs are definitely short of LHWs for HMA. On the other hand, LHWs are unstable because of the low salary *(LHWs on HMA in THC)*. LHWs are not enough and we have to arrange our time more appropriately, however, if the residents are not free on weekdays, we have to work on weekend, thus sometimes we can only have a day off at weekends *(LHWs on HMA in THC)*. There is short of personnel in THC, I’ll be easier if there were 2-3LHWs for HMA. I would be anxious while I provide health check by myself for more than 20 elderly at the same time *(LHWs on HMA in THC)*. There is lack of LHWs, for example, there are not enough health workers to provide health check for the elderly, moreover, we lack a radiologist and have to find part-time worker in other hospital now *(LHWs on HMA in THC)*. We (nurses) cannot guide the elderly patients on medicine intake because we have no doctors in our team of HMA while we (nurses) have relatively less knowledge and skills *(LHWs on HMA in THC)*. We need more general practitioners (GP). If there were no team of GP, nurses could not solve many health problems due to their insufficient professional knowledge and skills. The most in need now is professional GP *(Leader in CHC).*
External environment	In-coordinate cooperation Of multisectoral	Almost all LHWs and leaders reported less multi-sectors cooperation from broadcasting and television media, sub-district office, neighborhood committee, urban management, and community management in HMA publicity, assisting in informing elderly to take HC, health lectures and providing propaganda materials which resulted All LHWs reported PHCs need share information about medical services received by the elderly in hospitals. Moreover, some PHC sectors had poor internal cooperation between clinical and public health departments.	The propaganda is not enough, a lot of residents have no any information on HMA. They do not understand why you provide them health check for free, do not believe this work is good for them, and unwilling to leave their true ID or phone information, which make our work hard to carry out. We try to explain at first, but now we feel disappointed by residents’ misunderstanding *(LHWs on HMA in THC)*. In our CHC, the department of clinical medicine completely was separated from the department of public health, and clinicians are unwilling to support the work of public health *(LHWs on HMA in CHC)*. Poor coordination of community management and community health service. When we provide health service in community, the urban management officers and the community managers could not support us and even come to impede us *(LHWs on HMA in THC).* Many elderly have their own units which organize them to do regular physical examination in big hospitals. We hope to share the health information with the big hospitals, but now, we are totally separated with those big hospitals *(LHWs on HMA in THC).*
Implementation infrastructure	Shortage of equipment and medicine	Though infrastructure has improved in most of PHC sectors recent years, many LHWs reported outdated equipment and limited medicine in PHC sectors. Moreover, some leaders reported PHC sectors in city lacked of adequate space and barrier-free facilities like elevator, and some PHC sectors in rural area were located in area with underdeveloped transportation	There has traffic problem while we visit resident in rural area. Usually, when we get off the bus, we still have to walk at least a quarter of an hour to resident’s home, with the medical equipment in hand, which is definitely inconvenient *(LHWs on HMA in THC)*. Limited drugs and equipment in THC lead we cannot identify health problems of the elderly and provide therapy to their health problems *(LHWs on HMA in THC)*. It is incontinent for the elderly because we do not have elevators in the building *(Leader in CHC).*

Notes: BPHS refers to basic public health service; PHC refers to primary health care; CHCs refers to community health centers; THCs refers to township hospital centers; HMA refers to health management of the aged; LHWs refers to health care workers; HC refers to health check-up; HG refers to health guidance; FGDs refers to focus group discussions

#### Interventions

Firstly, the fixed HC items are unattractive to the aged people because the items could not meet the various needs of the aged people, so not all of the elderly were satisfied with the current HMA program. Secondly, the performance assessment was inappropriate because it mainly emphasized the universal coverage of HMA program (the rate of the elderly included in HMA program) assessing the quantity of the program, but it ignored the quality of the HMA program delivery. Thirdly, funds are insufficient not only for provision of HMA service but also for the salary payment of LHWs. Hence, that PHC sectors had to take “clinical support public health” measures (to improve salary for HCWs who deliver BPHS by using the revenue from the clinic department in PHC sectors) were reported by many leaders and HCWs.

#### Recipients

Firstly, poor health literacy among the elderly or their families was one of the main obstacles for the elderly to actively participate in HMA program. Secondly, PHC sectors are extremely short of fully-qualified LHWs to undertake HMA program.

#### External environment

The perceived external barrier was less multi-sector cooperation in HMA publicity and the organizing residents to receive HMA. Moreover, some LHWs reported poor internal cooperation between clinical and public health departments when delivering HMA. Accordingly, LHWs delivering HMA in PHC sectors were not only responsible for providing HMA service but also for organizing the elderly to participate HMA.

#### Implementation infrastructure

In recent years, the infrastructure has been improved in most PHC sectors, especially the buildings. However, outdated equipment and limited medicines in PHC sectors were still often reported. Some PHC sectors were located in inconvenient transportation areas, which was an obstacle for aged people to participate in HMA program.

## Discussion

In China, all items of HMA in BPHS are delivered by LHWs in PHC sectors. However, HMA delivery varies substantially among different regions. LE and HA, HC, AE and HG were the four items of HMA, we evaluated the achievements of HMA delivery by participants’ knowledge and utilization of, and satisfaction to each item of HMA. Regarding knowledge of HMA, 64.4% of aged individuals are familiar with HMA in Chengdu, Sichuan [51], while only 58.38% are familiar with HMA in Hubei [52]; both values are much lower than what we discovered. Our study also disclosed that at least 86% of aged individuals in Southwest China are willing to use HMA; which is much higher than 65.27% in Urumqi City [20]. More than 90% of aged individuals are satisfied with HMA in the Sichuan and Shandong provinces [13, 51], which is consistent with our study, while lower levels of satisfaction are found in Henan (49.3%) [27], Hubei (51.27%) [52] and Zhejiang (64.4%) [18] Provinces. We discovered that the knowledge, utilization and satisfaction of HMA are relatively higher in Southwest China compared to other regions [18, 20, 27, 51, 52]; especially the rate of satisfaction, which exceeds 94%.

Individual studies analyzed factors associated with HMA delivery found that the aged people in rural area were more likely satisfied with HMA program [15, 53], but another study reported that the aged people in urban area were more likely to use HMA [28]. Our results from multivariate logistic regression model similarly found that characteristic of the aged population, including gender, occupation and BMI as well as the quality of PHC sectors and economy of living place, were associated with knowledge, utilization and satisfaction to HMA. Therefore, more attention should be focused on the aged people from less developed area and in PHCs of good quality with overweight/ female/farmers aged people, when implementation of HMA. These results hinted the strategy to deliver HMA program, which need adapt to the aged population with different characteristics.

This study also confirmed that HMA delivery is confronted with various and complex challenges, despite that all PHC sectors carrying out HMA have made many achievements in Southwest China.

### Intervention

Foremost, the HMA item itself has several barriers. Firstly, the equalization of BPHS emphasizes providing BPHS that is responsive to residents’ needs rather than providing the same BPHS to everybody. Thus, the one-size health check-up items in HMA cannot fit all the needs of aged individuals with increasing health needs of the elderly according to the qualitative study. Furthermore, our study disclosed that PHC sectors delivered the one-size HC in Guizhou to aged individuals, which is not attractive enough and cannot meet aged individuals’ actual needs; similar outcomes have been reported by Zhao HF [54] and Li L [13]. Although HMA in a better economic region, such as Chongqing, increased several items of HC, it could still not meet all the needs. The WHO put forth “people-centered and integrated health service” in 2015 [55], which implied that the equalization of BPHS required a thorough and regular health needs assessment to detect the current health problems faced by local people, and the assessment can be used to design needs-based program. So, the HMA program design needs to continually update and conduct health needs assessment of aged individuals to be able to better respond to their health needs. Secondly, an appropriate performance assessment system is the key to ensure the orderly development of HMA items [28]. Actually, consistent with the findings of Hu XY [30], we discovered that the present performance assessment focusing more on the rate of aged individuals participating in HMA in Southwest China is contrary to the policy of optional participation by aged individuals. Hence, performance assessment could be modified to consider both coverage and quality of HMA. Thirdly, as for insufficient funds for the HMA program, which has been reported in Hainan province [53], the majority of PHC sectors in Southwest China adopt a method with the definition of “clinical support public health” (to improve salary for HCWs who deliver BPHS by using the revenue from the clinic department in PHC sectors), which is unfortunately far from enough. On the other hand, funding for LHW employment is insufficient in PHC sectors [56], which results in LHWs’ low income and potential of aggravating the plight of LHWs shortage.

### Recipients

Poor health literacy is one of the main barriers among aged individuals. Wen XQ et al. [57] suggested that improving the health literacy of aged individuals could significantly increase the effective utilization of HMA and promote their health. Our study disclosed that some aged residents are reluctant to participate in HMA because they do not recognize the importance of prevention, and thus consider it was unnecessary to take the same check-up every year, which is similar to the report by Shi FF [16] in Chengdu. Furthermore, we found that some aged people are unwilling to encounter the pressure of diseases and associated economic burden, similar to the report by Jiang RQ [22]. In addition, our study also found that in spite of possessing health literacy, some aged people still fail to access HMA for the poor health awareness of their family.

Furthermore, PHC capacity is the driving force of the quality of HMA. Although with great emphasis from the central government PHC sectors in China have developed rapidly since 2006, our study indicated that a low capacity of PHC is a key barrier to deliver qualified HMA. According to the people-centered model, LHWs should provide service that is responsive to the needs and expectations of older people [58, 59]. To achieve this goal, PHC sectors must have a multidisciplinary LHW team to provide integrated care with good quality [1]. The team members must have strong professional competencies and take full advantages of these talents. In addition, it is important to ensure that LHWs have basic gerontological knowledge and skills, as well as general competencies to work in integrated systems, including communication, teamwork and other skills [1]. Unfortunately, we found that all PHC sectors in Southwest China lack enough qualified LHWs. And LHWs for HMA often undertake multiple BPHS items with heavy workloads. Similarly, Liang XH [31] discovered that there is still a substantial shortage of LHWs for BPHS in Chongqing. Additionally, Yang L et al. [60] revealed that with increasing BPHS items, sufficient LHWs are not equipped simultaneously, which exacerbates the shortage of LHWs in PHC sectors. Furthermore, heavy workload and low income results in unstable work teams in PHC sectors [55]. We found that PHC sectors are extremely short of general practitioners and that the current LHWs for HMA often have low education level and no related professional background, which is consistent with the findings of Cai XN [55] in Haikou. Meanwhile, Li W et al. [61] confirmed that there are currently “four low levels” of “low education, low professional ability, low technical title and low salary” among LHWs delivering BPHS in Chongqing. Healthcare workers with high education and professional titles are not willing to work in PHC sectors with low salaries, which may further lead to the loss of valuable LHW teams. Remarkably, limited knowledge prevents LHWs from providing efficient and qualified HMA for aged individuals.

### External environment

Regarding the external environment, though we find 95% aged people know about HMA program, but qualitative results indicated the poor literacy of the elderly is the reason for them to inactively participate in HMA program, which has been consistently reported by Yu L [14] and Wei YL [51] in other cities. Our study disclosed that the utilization of HMA could be improved with support from the families of aged individuals. In China, PHC sectors are not well recognized by residents. All BPHS programs delivered by PHC sectors including HMA need multi-sector cooperation to disseminate the program information to residents, and to organize residents to participate in the programs, or to share health information. However, this study found the multi-sector cooperation or whole social participation in HMA is of great essentials and need to be attached more importance. We found that current propaganda work on BPHS only depends on PHC sectors themselves, whose work is far from enough to have aged individuals, their families or our society as a whole to accept the HMA program. The government and media fail to take their roles in HMA propaganda. LHWs do not receive support from sub-district offices, neighborhood committees, urban management, community management or superior hospitals during the implementation of HMA. PHC sectors work independently to provide HMA, resulting in difficulties in obtaining support and recognition from aged individuals and others.

The disease-based curative models should transform to the provision of older-person-centered integrated care [1]. “Integrated care” ensures that people receive a continuum of health promotion, disease prevention, diagnosis, treatment, disease management, rehabilitation and palliative care services at different levels and sites of care within the health system, according to their needs throughout their whole life [54], which is indispensable from multi-sector cooperation. However, it is difficult to achieve “integrated care” only by contacting and coordinating PHCs. The joint effort of departments in PHC sectors and the cooperation between departments of social security and health care are necessary to further improve work efficiency and effectiveness of HMA programs.

### Implementation infrastructure

Finally, insufficient materials and outdated equipment are common in PHCs [31]. Although hardware conditions in PHCs have improved recently, there still exist problems such as lack of space, barrier-free facilities shortages and poor transportation in rural areas [62]. Due to outdated equipment (for example, no computed tomography in most PHCs), some aged distrust the medical examination results, which may reduce their enthusiasm in the participation of HMA. The lack of common medicines, such as drugs for chronic diseases, have the aged individuals unable to enjoy “one-stop service” in PHC sectors, which also fortifies the difficulty of HMA implementation.

### Strengths and limitations

A previous study on HMA in BPHS focused on the rate of HMA provision in PHCs [24–27], aged residents’ knowledge, and utilization or satisfaction with HMA [14–24, 63, 64], and only a few studies have explored the barriers in PHCs to implement HMA [28–30, 32, 55]. Our study is the first step towards a systematic assessment of HMA provision in Southwest China to discover the main challenges and provide further constructive countermeasures. However, we did not include policymakers from the local Health and Family Planning Commission, as our study focused on participants who could provide information about their difficulties of and suggestions on addressing barriers faced by PHC sectors, along with participants of a questionnaire survey who were recruited from PHC sectors rather than from the community. The aged people included in this study were recruited from PHCs and may be more likely to trust PHC sectors and be more accessible to HMA service than those who were excluded; thus, the rates of knowledge, utilization and satisfaction to HMA of study participants could be higher than those of the overall aged community, which would be a bias for questions on knowledge and utilization of, and satisfaction on HMAs in the structured interview.

### Implications

HMA is aimed at the early detection of risk factors, controlling disease and promoting healthy ageing. The findings from this study may not generalize to other provinces in Central and Eastern China, where socio-economy and PHC sectors have developed better compared to those of Southwest China; however, based on evidence from this study, public health strategies can be taken on by regions in Western China or countries with the same socio-economic characteristics to promote healthy ageing. For each specific context, the exact mix of strategies can be developed, with local contexts, values and preferences taken into account [1].

Foremost, a comprehensive assessment with fully understanding the situation in each PHC, optimizing the performance assessment strategy and giving priority to performance appraisal orientation of the health needs of local aged is imperative for designing older-person-centered HMA programs. Secondly, sufficient funding is the precondition for the implementation of HMA in all fields, such as health demands assessment, health literacy improvement, and strengthening software and hardware in PHCs. Thirdly, by increasing and reinforcing government and media roles in the propaganda of HMA programs, the participation and self-care capacity will be further improved. Moreover, better health literacy could guarantee more real and effective results of health needs assessment, which is the main factor for the knowledge, utilization and satisfaction of HMA as well [64]. Fourthly, the development of LHWs’ competency should be at the center of capacity building in PHC sectors. Emphasizing in-service training and continuing professional development are essential for consolidating knowledge and upgrading skills for LHWs. Some strategies and incentives such as the introduction of a pension program, support for professional promotion, and bonuses [65] are also essential to attract qualified LHWs. Last but not least, hardware capacity and more investment from the government are needed to reinforce basic infrastructure construction and extend the basic medication directory to narrow the gap with tertiary hospitals, in particular, to increase the amount of drugs needed to treat common diseases among aged individuals in PHCs.

## Conclusions

Acceleration of ageing brings unprecedented challenges to the health system in developing countries such as China. The Chinese BPHS program has paid great attention to the health care of aged individuals, and HMA in BPHS has achieved progress, as demonstrated by the fact that the knowledge on and utilization of, satisfaction to HMA of aged people was higher in recent years in Southwest China compared with other regions in past years. However, HMA in this region is facing challenges in further quality improvement. The “older-person-centered and integrated care” model provides a sound and profound theoretical basis to address those challenges, including reinforcing the needs-based HMA design, building the capacity of PHC sectors, improving the LHWs’ competency as well as strengthening multi-sector cooperation.

## Figures and Tables

**Fig. 2:**
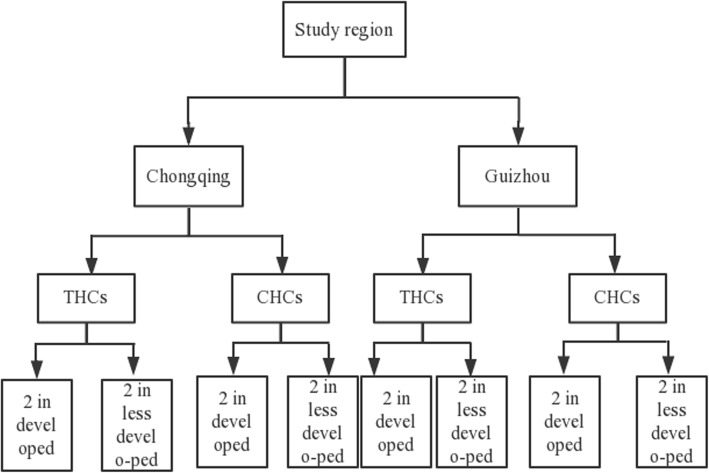
Flow chart of study region selection

## Data Availability

The datasets used and/or analyzed during the current study are available from the corresponding author on reasonable request.

## Appendix B

**Table 5:** Chi-square analysis on factors associated with knowledge and utilization of, and satisfaction to HMA

Variables	Knowledge				Utilization				Satisfaction			
	LE and HA	HC	AE	HG	LE and HA	HC	AE	HG	LE and HA	HC	AE	HG
Age												
60-70	369(87.4)	405(96.0)	399(94.5)	378(89.6)	360(88.9)	393(95.2)	389(94.4)	367(89.7)	347(96.4)	373(94.9)	371(95.4)	352(96.2)
70-80	220(81.5)	260(96.3)	260(96.3)	238(88.1)	215(84.3)	255(95.5)	255(95.5)	233(89.3)	199(92.6)	233(91.4)	232(91.0)	217(93.1)
≥80	43(75.4)*	54(94.7)	54(94.7)	49(86.0)	42(76.4)*	50(87.7)*	50(87.7)	47(82.5)	42(100.0)*	50(100.0)*	50(100.0)*	47(100.0)
Gender												
Male	242(85.2)	271(95.4)	269(94.7)	254(89.4)	235(87.0)	263(94.9)	261(94.2)	244(89.1)	219(93.2)	242(92.0)	242(92.7)	226(92.6)
Female	389(83.8)	447(96.3)	443(95.5)	410(88.4)	381(85.8)	434(94.6)	432(94.3)	402(88.9)	368(96.6)*	413(95.2)*	410(94.9)	389(97.0)*
Residence												
Rural	243(85.0)	278(97.2)	277(96.9)	256(89.5)	240(89.2)	271(96.4)	271(96.8)	253(91.7)	228(95.0)	260(95.9)	260(95.9)	244(96.4)
Urban	383(84.0)	434(95.2)	429(94.1)	402(88.2)	372(84.5)	421(93.8)	417(92.9)*	388(87.4)	356(95.7)	391(92.9)	388(93.0)	367(94.8)
Region												
Chongqing	479(90.2)	510(96.0)	507(95.5)	495(93.2)	467(94.0)	496(95.6)	494(95.4)	484(95.1)	443(94.9)	470(94.8)	470(95.1)	461(95.2)
Guizhou	153(70.2)*	209(95.9)	206(94.5)	170(78.0)*	150(68.8)*	202(92.7)	200(91.7)	163(74.8)*	145(96.7)	186(92.1)	183(91.5)	155(95.7)
Type of PHCs												
THCs	397(85.2)	447(95.9)	442(94.8)	415(89.1)	392(89.3)	435(95.4)	432(94.9)	407(90.8)	373(95.2)	413(94.9)	413(95.6)	391(96.1)
CHCs	235(83.0)	272(96.1)	271(95.8)	250(88.3)	225(81.5)*	263(93.6)	262(93.2)	240(86.2)*	215(95.6)	243(92.4)	240(91.6)*	225(94.1)
Quality of PHCs												
Good	329(78.9)	395(94.7)	390(93.5)	353(84.7)	324(80.6)	381(93.2)	378(92.6)	343(84.9)	306(94.4)	355(93.2)	353(93.4)	325(95.0)
Poor	303(91.3)*	324(97.6)*	323(97.3)*	312(94.0)*	293(93.6)*	317(96.6)*	316(96.3)*	304(94.1)*	282(96.2)	301(95.0)	300(94.9)	192(95.7)
Marital status												
Married	471(84.6)	534(95.9)	530(95.2)	496(89.0)	460(86.5)	518(95.0)	515(94.7)	481(89.6)	440(95.7)	490(94.6)	487(94.6)	459(95.4)
Divorced/Widowed	156(83.4)	180(96.3)	178(95.2)	164(87.7)	153(86.0)	176(94.1)	175(93.6)	162(87.6)	145(94.8)	163(92.6)	162(92.6)	153(95.0)
Education												
Primary and below	396(84.3)	448(95.3)	444(94.5)	415(88.3)	389(87.4)	437(95.0)	434(94.6)	406(89.8)	371(95.4)	417(95.4)	415(95.6)	390(96.1)
Middle school	157(86.7)	176(97.2)	175(96.7)	164(90.6)	152(85.9)	169(93.9)	169(93.9)	158(88.8)	145(95.4)	154(91.1)	155(91.7)	149(94.3)
College and above	78(80.4)	94(96.9)	93(95.9)	85(87.6)	75(81.5)	91(94.8)	90(93.8)	82(85.4)	71(94.7)	84(92.3)	82(91.1)	76(93.8)
Occupation (*n*=765)												
Employed in enterprises/institutions/government	227(82.8)	261(95.3)	260(94.9)	241(88.0)	217(81.6)	252(93.0)	251(92.6)	230(85.2)	206(94.9)	232(92.1)	229(91.2)	213(93.0)
Peasants/ rural migrant workers	220(82.1)	255(95.1)	251(93.7)	231(86.2)	216(88.5)	246(94.3)	243(93.1)	226(89.0)	211(97.7)	237(96.3)	236(97.1)	221(97.8)
Others	178(89.0)^#^	196(98.0)	195(97.5)	186(93.0)^#^	177(89.4)*	193(97.5)	193(98.0)*	184(93.9)*	167(94.4)	182(94.3)	183(94.6)*	177(96.2)*
Health insurance												
Basic health insurance	620(84.4)	705(95.9)	699(95.1)	652(88.7)	605(86.2)	685(94.7)	681(94.3)	634(88.8)	576(95.2)	644(94.0)	641(94.1)	604(95.4)
No Basic health insurance	8(88.9)	9(100.0)	9(100.0)	9(100.0)	8(88.9)	9(100.0)	9(100.0)	9(100.0)	8(100.0)	8(88.9)	8(88.9)	8(88.9)
No health insurance	3(75.0)	4(100.0)	4(100.0)	3(75.0)	3(100.0)	3(75.0)	3(75.0)	3(75.0)	3(100.0)	3(100.0)	3(100.0)	3(100.0)
Distance to PHCs												
<1km	493(84.4)	562(96.2)	557(95.4)	521(89.2)	481(86.2)	548(94.8)	544(94.3)	508(89.0)	461(95.8)	519(94.7)	516(94.9)	489(96.3)
1-2km	71(79.8)	84(94.4)	83(93.3)	75(84.3)	72(85.7)	82(95.3)	82(95.3)	76(90.5)	68(94.4)	75(91.5)	75(91.5)	70(93.3)
≥2km	63(88.7)	68(95.8)	68(95.8)	64(90.1)	59(86.8)	63(92.6)	63(92.6)	58(86.6)	54(91.5)	57(90.5)	57(90.5)	53(91.4)
BMI(kg/m2)												
<18.5	20(62.5)	30(93.8)	29(90.6)	25(78.1)	20(66.7)	30(96.8)	29(93.5)	25(88.9)	20(100.0)	29(96.7)	27(93.1)	25(100.0)
18.5-24.0	377(84.3)	423(94.6)	422(94.4)	394(88.1)	367(86.4)	411(93.6)	411(93.8)	383(80.6)	349(95.1)	383(93.2)	382(92.9)	363(94.8)
24.0-30.0	187(86.6)	212(98.1)	208(96.3)	196(90.7)	182(87.9)	203(95.3)	200(93.9)	189(89.6)	173(95.1)	191(94.1)	191(95.5)	179(95.2)
≥30.0	39(95.1)*	41(100.0)	41(100.0)	40(97.6)*	39(97.5)*	41(100.0)	41(100.0)	40(97.6)	37(94.9)	40(97.6)	40(97.6)	39(97.5)

**P*<0.05

## Abbreviations

AE: Auxiliary Exam
BPHS: Basic Public Health Service
CHC: Community Health Center
FGD: Focus group discussion
HC: Health Check-up
HG: Health Guidance
HMA: Healthcare Management for the Aged
LE and HA: Lifestyle Evaluation and Health Assessment
LHWs: Lay Healthcare Workers
NCDs: Non-Communicable Diseases
PHC: Primary Health Care
PRISM: Practical Robust Implementation and Sustainability Model
SPSS: Statistical Package for Social Science
THC: Township Hospital Center
